# Induction of molecular remission by using anti-CC-chemokine receptor 4 (anti-CCR4) antibodies for adult T-cell leukemia: a risk of opportunistic infection after treatment with anti-CCR4 antibodies

**DOI:** 10.1186/1742-4690-11-S1-P15

**Published:** 2014-01-07

**Authors:** Yasuhiuro Maeda, Go Eguchi, Takahiro Kumode, Terufumi Yamaguchi

**Affiliations:** 1 Department of Hematology, National Hospital Organization Osaka Minami Medical Center, Osaka, Japan

## 

The CC-chemokine receptor 4 (CCR4) is expressed in almost ATLL cells. Thus, anti-CCR4 antibodies can be used as a treatment strategy for ATLL. Mogamulizumab (MOG), which is a defucosylated anti-CCR4 monoclonal antibody, showed good results even in patients with recurrent ATLL in phase I or II studies. We treated 8 elderly patients with ATL who were resistant to chemotherapy using MOG monotherapy. All patients received 1.0 mg/kg of mogamulizumab (MOG) once per week for 8 weeks by intravenous infusion. In the present study, we observed CCR4-specific ADCC against CCR4-positive ATL cells. All patients showed CR with a marked decrease in the number of ATL cells. However, 2 patients contracted viral infection because of severe lymphopenia. One patient died because of severe cytomegalovirus infection despite adequate treatment. One patient had Stevens’ Johnson syndrome. These results suggested that MOG was effective in chemotherapy-resistant ATL patients. However, CCR4 is not only on ATL cells but also on endogenous Treg. The decrease in the number of Treg after MOG monotherapy has been expected to boost the antitumor activity and to be involved in the development of immune disorders, including autoimmune diseases. Furthermore, a decrease in CD4+ T cells led to viral infection. In conclusion, several treatments for prophylaxis of opportunistic infection, including CMV infection, should be recommended.

